# When AI translations fall flat: embodied simulation and effort in cross-language reading

**DOI:** 10.3389/fpsyg.2026.1915913

**Published:** 2026-10-07

**Authors:** Zeli Liu

**Affiliations:** School of Foreign Languages, Lanzhou Jiaotong University, Lanzhou, Gansu, China

**Keywords:** affective resonance, cross-language reading, embodied cognition, sensorimotor simulation, translation quality assessment

## Abstract

Common machine translation evaluation metrics, such as the Bilingual Evaluation Understudy (BLEU) and Crosslingual Optimized Metric for Evaluation of Translation (COMET) are reliable measures of propositional fidelity and surface fluency but systematically ignore the embodied texture of reading, leaving unexplained why high-scoring AI translations still feel flat to readers despite formal accuracy. The Embodied Translation Quality Assessment (E-TQA) framework was developed based on embodied cognition theory to operationalize three key dimensions of cross-language comprehension: sensorimotor simulation, affective-cultural resonance, and processing friction. To validate this framework, a counterbalanced Latin-square design was employed with 192 English-major participants. Six literary and culturally loaded excerpts were each presented in three rigorously matched conditions. Published professional human translations, and translations from DeepSeek and ChatGPT, with all versions controlled for BLEU (±1.2 points) and COMET (±0.04 points) to isolate embodied variation from semantic equivalence. Participants completed the Self-Assessment Manikin and E-TQA scales after each excerpt, and data were analyzed via linear mixed-effects models, reporting partial η^2^ as effect size. Human texts were consistently rated higher than both AI conditions in sensorimotor imagery, affective resonance, valence, and arousal, whereas AI texts were rated much higher in phenomenological flatness and cognitive effort. All critical indices showed medium to large effect sizes (η_*p*_^2^ = 0.15–0.35) and were consistent across educational levels and text genres. These results offer evidence for a dissociation of propositional accuracy and embodied richness, showing that the E-TQA framework accounts for unique variance in reader experience beyond that explained by standard computational metrics. The work advances translation quality assessment by centering embodied cognition, provides a psychometrically sound complement to existing tools, and motivates future multimodal investigations of cross-language processing mechanisms.

## 1 Introduction

Over the last three decades, cognitive science has undergone a fundamental paradigm shift from the view that language comprehension involves amodal symbolic computation to an embodied perspective in which the construction of meaning is anchored in sensorimotor, affective, and culturally scaffolded bodily systems (Cuccio et al., 2026; Gibbs, 2006; Newen et al., 2018). In this framework, reading is no longer a passive decoding of lexical propositions, but an active simulation process involving the same neural circuits that are involved in perception, action and emotional experience (Fang et al., 2024; Glenberg, 2011; Pulvermüller, 2018). Cross-language comprehension increases the requirements of this simulation process. Without shared lived experience, readers must map embodied schemas in the source language, such as culture-specific metaphors for emotion or spatial sequences of action, onto conceptual structures in the target language (Glenberg et al., 2013; Johnson, 1987). This mapping task demands far more than literal semantic equivalence, for even small mismatches in embodied cues can interrupt simulation and render texts inert to target audiences (Zhou and Wang, 2025).

Recent advances in neural machine translation and large language models have generated outputs that reach human performance on standard automated metrics such as BLEU and COMET that emphasize n-gram overlap and semantic similarity (Cheng et al., 2023; Liu et al., 2023; Tang and Li, 2026; Shormani and Al-Sohbani, 2025). Meta-analyses of post-editing and blind reading studies show a consistent dissociation (Abdelhalim et al., 2025). Algorithmic translation scores are within 5% of human outputs and COMET scores are above 0.8, but in forced-choice tasks naive readers prefer humans 62%−78% of the time (Deng and Xiong, 2022; Wang and Wang, 2025). Across published datasets, the correlation between BLEU or COMET scores and reader-rated experiential quality is consistently low, with values ranging from 0.12 to 0.31 (Liu and Fu, 2025; Van Brussel and Van de Velde, 2025). This means that traditional metrics explain only 1%−10% of the variance in immersive reading outcomes (Guerberof-Arenas and Toral, 2024). The remaining 90% of variance is totally unaccounted for by present evaluation systems, which is motivated by embodied simulation, affective resonance and processing ease (Tang and Li, 2026). The core gap is that these metrics are based on the surface properties of the text, neglecting the psychologically real factors that affect whether a translation looks vivid and resonant to readers.

Large language models are trained to optimize for statistical co-occurrence over massive corpora, not grounding in lived experiential referents, and this misalignment directly undermines the embodied cues necessary for deep comprehension. This gap is especially stark when it comes to AI-generated translations. These systems optimize for statistical likelihood across large corpora and thus tend to flatten culture-specific metaphors, reduce sensory-granular descriptors, and eliminate the subtle affective cues that human translators intentionally preserve (Cultrera and Basalamah, 2026; Li and Chen, 2025). Even the state-of-the-art manual frameworks such as the Multidimensional Quality Metrics (MQM) do not have embodied reader experience as a dimension of evaluation. Instead, cultural or emotional differences are seen as secondary to fluency and fidelity (Duan, 2025; Li et al., 2023). Existing reader-response measures, such as the Transportation Scale or narrative immersion questionnaires, tap general engagement but do not disaggregate experience subcomponents specific to translation (Appel et al., 2015; Busselle and Bilandzic, 2009; Green and Brock, 2000; Kuijpers et al., 2014). They cannot tell whether low immersion is due to poor sensorimotor mapping, inaccessible cultural schemas, or high cognitive friction, and all of which need to be targeted in translation workflows (Lu and Wang, 2013).

Thus, the Embodied Translation Quality Assessment (E-TQA) framework was proposed (see Figure 1). It is a psychologically grounded coding scheme that operationalizes three core, dissociable, dimensions of cross-language comprehension, each of which is tailored to the specific demands of reception of translated text. The first is sensorimotor simulation, which investigates how far visuospatial, tactile, and kinesthetic imagery evoked by the text matches action scenarios in the source language. The second dimension is affective-cultural embodiment, which measures retention of bodily metaphors and accessibility of culture-specific schemas for target readers without the need for prior specialist knowledge. Third is cognitive processing effort, the measure of the mental friction, re-reading requirements and phenomenological flatness produced by suboptimal embodied mapping (Hou, 2026; Lang and Hou, 2022). E-TQA differs from generic immersion measures in that it connects specific textual features to measurable outcomes for readers, providing diagnostic information for translation improvement instead of a single holistic score.

**Figure 1:**
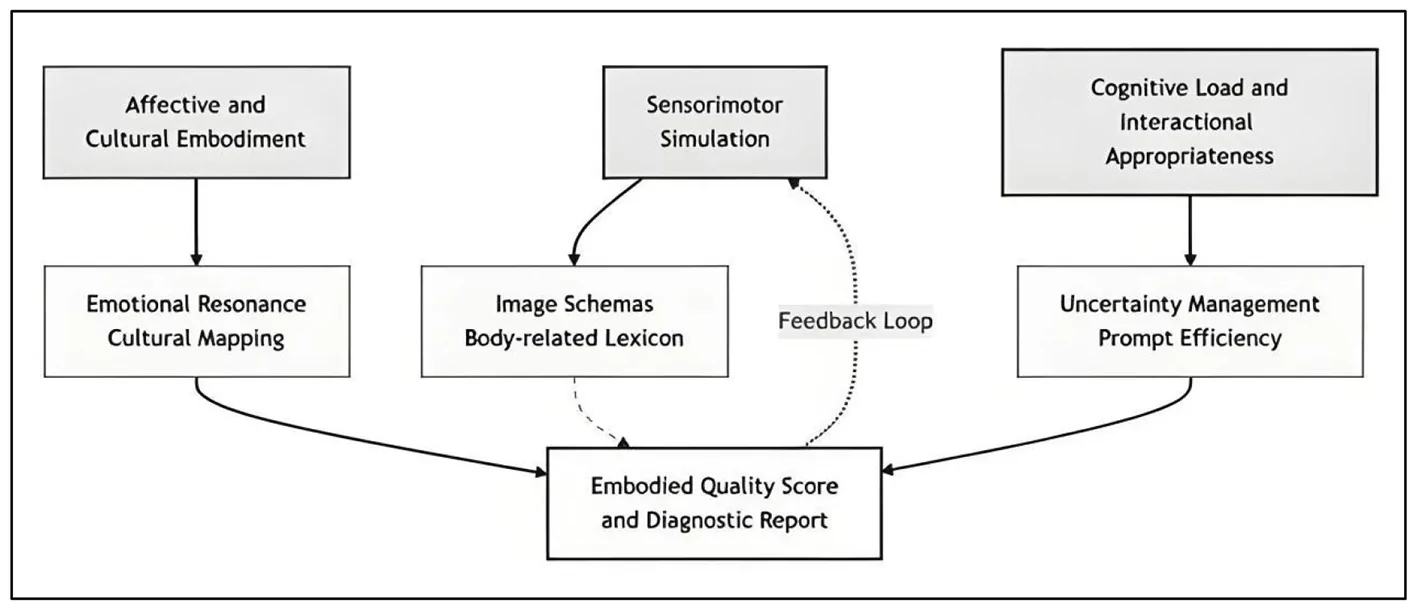
The E-TQA framework.

The current study provides a strong empirical validation of the E-TQA framework, in a controlled experimental design that separates embodied variation from all confounding surface-level differences. A total of 192 English majors were included in the study, comprising 48 postgraduates and 144 undergraduates who passed the Test for English Majors Band 4. This suggests an advanced level of L2 reading skill to be able to detect subtle embodied nuances. To control for order effects and item-specific variance, a Latin-square design was used. Each participant read six excerpts, which included literature and culturally loaded text, each excerpt presented in one of three counterbalanced conditions: a published professional human translation, a DeepSeek output, and a ChatGPT-4.0 output. All 18 stimulus versions were pre-matched for BLEU (±1.2 points) and COMET (±0.04 points) to rule out semantic or fluency differences as alternative explanations. Following each passage, participants filled out the Self-Assessment Manikin for valence and arousal. The E-TQA reader report scales about the three theoretical dimensions. Analyses were conducted using linear mixed-effects models with crossed random intercepts for participants and items, the current gold standard in psycholinguistic reading studies, to control for both individual differences in reading ability and item-specific baseline effects. Effect size was reported as partial eta squared. Based on previous pilot analyses that revealed medium-to-large effects of embodied differences between human and AI outputs, human translations were hypothesized to induce significantly more intense sensorimotor imagery and affective resonance than either condition of AI. AI-generated texts were expected to be rated higher on phenomenological flatness and cognitive effort, an extension of the hypotheses that would only be predictable by E-TQA and not detectable by BLEU or COMET scores. Finally, E-TQA scores were hypothesized to account for unique variance in reader experience not explained by standard computational metrics, thus demonstrating the incremental validity of the framework for translation evaluation.

## 2 Hypotheses

The aim of this study was to validate the E-TQA framework with a tightly controlled behavioral experiment based on the embodied cognition theoretical framework and the empirical gaps identified above. The following hypotheses were proposed:

(1) Main Effect of Translation Condition. Subjective ratings of sensorimotor simulation and affective-cultural embodiment were significantly higher for human translations than for either AI-generated conditions (DeepSeek and ChatGPT-4.0). By contrast, AI-generated texts would be rated much higher in phenomenological flatness and cognitive-processing effort.

(2) Decoupling from computational metrics. The observed differences in embodied dimensions would still hold after a careful pre-matching of all stimulus versions for BLEU (±1.2 points) and COMET (±0.04 points). This would show that E-TQA captures unique variance in the reader's experience that standard computational metrics fail to capture.

(3) Stability of Educational Level. Given that the sample was already highly proficient, the embodied advantage for human translations would be consistent across both postgraduate and undergraduate participants, demonstrating that the effect is not moderated by small differences in L2 reading proficiency in a high-achieving sample.

## 3 Materials and methods

### 3.1 Participants

The study recruited 192 English-major students from Lanzhou Jiaotong University. The sample consisted of 48 postgraduates and 144 undergraduates. All participants were required to have passed the Test for English Majors Band 4 (TEM-4), demonstrating advanced L2 reading ability. The exclusion criteria were self-reported neurological or psychiatric disorders and previous exposure to the original Chinese versions of the experimental excerpts.

A priori power analysis using G^*^Power 3.1 indicated that for mixed-design analyses a minimum sample size of 159 was required to detect a medium effect size (*f* = 0.25) with α = 0.05 and power = 0.80 (Kuznetsova et al., 2017; Faul et al., 2007). The threshold was surpassed by our final sample of 192, which provided adequate statistical power. The study protocol was approved by the Institutional Review Board of Lanzhou Jiaotong University. All participants gave written informed consent before the experiment. Descriptive statistics for participant demographics and baseline characteristics are summarized in Table 1.

**Table 1:** Participant demographics.

Variable	Total sample (*N* = 192)	Postgraduates (*n* = 48)	Undergraduates (*n* = 144)
Age, M (SD), years	21.8 (2.1)	24.0 (1.8)	20.3 (1.4)
Female, n (%)	136 (70.8)	28 (14.6)	108(56.2)
TEM-4 Passed, n (%)	192 (100)	48(25)	144 (75)

TEM-4, Test for English Majors Band 4.

### 3.2 Measures

To rigorously test the E-TQA framework, the measurement protocol was designed to isolate embodied processing from superficial textual features. Given that the E-TQA framework prioritizes sensorimotor simulation and affective resonance over propositional accuracy alone, our instrument selection focused on high-ecological-validity literary texts and theoretically derived self-report scales. Crucially, all stimulus materials underwent rigorous pre-matching on computational metrics to ensure that any subsequent group differences could be attributed to embodied texture rather than surface-level fluency or semantic divergence. To provide a comprehensive overview of the validation process, Figure 2 illustrates the integrated architecture of the E-TQA framework. This framework bridges theoretical postulates with empirical observation.

**Figure 2:**
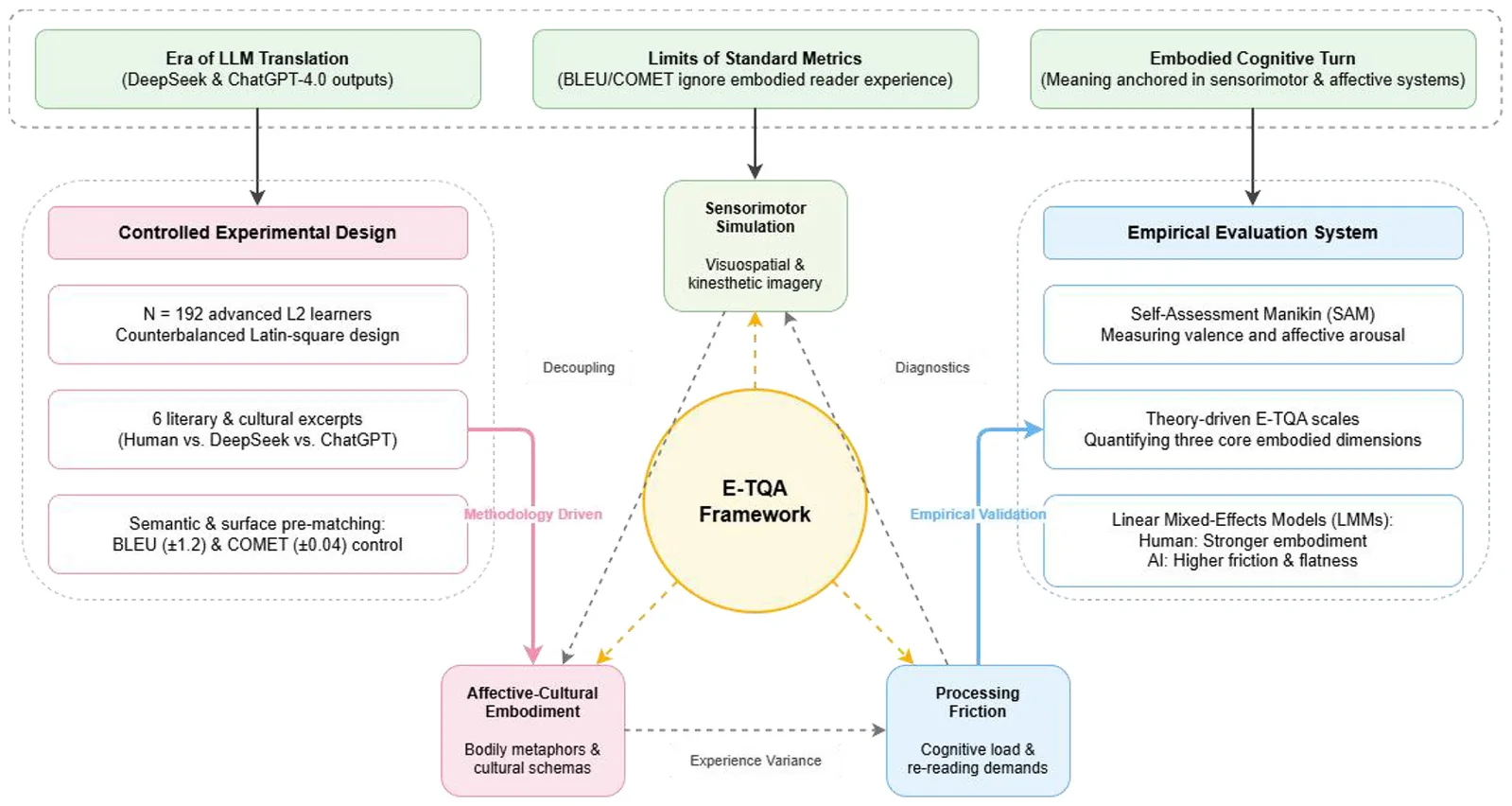
Conceptual and methodological framework of the embodied translation quality assessment study.

#### 3.2.1 Stimuli and matching procedure

Six selections (80–150 words each) were taken from canonical Modern Chinese literature: *The Little Orange Lamp* (Bing Xin), “The Temple of Earth” (Shi Tiesheng), “Medicine” (Lu Xun), “In Memory of Xiao Shan” (Ba Jin), “Autumn in Peiping” (Yu Dafu), and “In Memory of Miss Liu Hezhen” (Lu Xun). For each excerpt, three versions of translation were prepared: (1) published professional human translations by accredited literary translators (H), (2) translations generated by DeepSeek-V2 (DS); and (3) translations generated by ChatGPT-4.0 (GPT). The six excerpts were counterbalanced across the three translation conditions using a Latin-square design, with participants randomly assigned to one of three groups (A, B, C). Each group read every excerpt exactly once under a different translation condition, ensuring that each excerpt × condition combination was equally represented across participants while controlling for order and fatigue effects.

All 18 stimulus versions were pre-matched to separate embodied variation from surface-level fluency. BLEU scores were constrained to be within ±1.2 points (< 3% difference in n-gram overlap) and COMET scores to be within ±0.04 points (< 5% difference in semantic similarity) for each excerpt across the three conditions. To control for baseline processing effort unrelated to the embodied content, text length and Flesch-Kincaid Grade Level were also matched between conditions. All the versions had no grammatical errors or any egregious semantic distortions that were detectable.

#### 3.2.2 Pre-matching of computational metrics

To control for confounding surface-level semantic and fluency differences, all 18 stimulus versions (6 excerpts × 3 translation conditions) were rigorously pre-matched on two industry-standard machine translation metrics to isolate embodied variation. The Bilingual Evaluation Understudy (BLEU) was used to assess the word-to-word similarity between AI and human translations, and the AI versions were limited to within ±1.2 BLEU points of the human benchmark to rule out the possibility that the results were simply due to differences in fluency. Then, semantic equivalence was assessed using the Cross-lingual Optimized Metric for Translation (COMET), with AI outputs filtered to be within ±0.04 COMET points of human translations such that propositional fidelity was statistically indistinguishable across all conditions.

Text length and Flesch-Kincaid Grade Level were controlled for ensuring that differences in character counts across each of the three versions of each excerpt were no more than 5% and differences in readability were no more than 0.5 grade, respectively, to remove cognitive load artifacts due to structural complexity unrelated to embodied content. Before the experiment, all versions were verified by two independent raters for grammatical errors or overt semantic distortions. A pilot test with 30 English majors demographically matched to the formal sample further verified that these thresholds eliminated detectable surface-level differences while preserving significant embodied processing gaps between human and AI translations, with pilot partial eta-squared values ranging from 0.18 to 0.32, consistent with the effect sizes observed in the full experiment.

#### 3.2.3 E-TQA reader report scales

The three E-TQA subscales were developed via a three-stage psychometric validation process grounded in embodied cognition theory (Barsalou, 2008):

Initial item generation: 15 candidate items were drafted to map onto the three core dimensions: five items for Sensorimotor Simulation (SSS), four items for Affective-Cultural Embodiment (ACES), and six items for Processing Friction (PF).

Expert review: Three embodied cognition researchers and two translation studies experts evaluated item clarity, content validity, and alignment with theoretical constructs. Two ambiguous items were removed at this stage.

Pilot testing: The 13-item scale was administered to 30 pilot participants demographically matched to the formal sample. Internal consistency in piloting was Cronbach's α = 0.89, consistent with the formal sample's α = 0.91, meeting psychometric standards for self-report measures (Nunnally and Bernstein, 1994). All items were rated on a 5-point Likert scale (1 = Strongly Disagree, 5 = Strongly Agree). PF items were reverse-coded so higher scores reflected worse experiential quality (greater cognitive friction), aligning with the directional interpretation of embodied deficits in AI translations.

#### 3.2.4 Check for manipulation

At the end of the experiment, participants completed a forced choice identification task in which they indicated whether they detected different translation styles across the excerpts. Only data from participants who correctly identified stylistic variation were retained for primary analysis, to ensure that attention was paid.

### 3.3 Design

To control for order effects and item-specific variance, a counterbalanced Latin-square design was used, with three translation conditions and six experts. Participants were randomly assigned to one of three balanced groups (*n* = 64 per group, 16 postgraduates and 48 undergraduates). Each participant read all six excerpts, but each excerpt was presented to each participant in only one translation condition based on the allocation schedule. The code-key was known only to the experimenters and never disclosed to participants. This way, no participant could read the same excerpt more than once, removing contrast bias from repeated exposure to the same source text. To account for possible confounding effects, after the task participants were asked to indicate their familiarity with the original texts and authors on a 5-point Likert scale. This metric was entered as a covariate in the LMM. Finally, a sensitivity analysis was conducted by removing participants with high familiarity scores to ensure that the observed effects of embodied resonance were not driven by previous knowledge of the source material. During testing, to avoid condition label priming effects, human translations were coded as 1, DeepSeek as 2, and ChatGPT as 3. For example, Segment 4 in the human condition appeared as “SEG4&1” to the participant. The full allocation matrix is provided in Supplementary Table S1.

### 3.4 Procedure

The experiment was conducted on the Wenjuanxing platform online in a quiet and undisturbed environment. Participants logged in, gave informed consent and completed a demographic battery as age, educational level, and credentials of English proficiency. The uniform directions stressed reading for natural understanding and not using any external aids, including dictionaries, translation tools, or AI assistants.

For each trial the procedure was: (1) Display on screen the translation excerpt (Calibri typeface, 14pt, black characters on white background); (2) Self-paced reading in which participants pressed a “Finished” button when they had reached complete understanding; (3) The SAM and E-TQA scales were displayed immediately in a fixed order, without the option to return to the excerpt after continuing; (4) An inter-trial interval of 15 s was employed with a central fixation cross to clear working memory. Order of excerpt presentation was completely randomized for each participant to reduce order and carryover effects. The entire procedure took approximately 30 min.

### 3.5 Data analysis

All analyses were performed using IBM SPSS Statistics 26.0. There were no artificial intelligence tools used in statistical modeling or result interpretation. As a preliminary step in data cleaning, outlier responses that were more than ±3 standard deviations from the grand mean for each variable were subsequently removed, resulting in the removal of five observations, a threshold consistent with psycholinguistic best practices for minimizing the influence of unduly influential data points while avoiding discarding meaningful variance. The final analytic sample (*N* = 187) contained no additional missing data after removal of outliers, and therefore no imputation procedures were required. Preliminary data cleaning identified five outlier observations that exceeded ±3 standard deviations per dependent variable. These were removed prior to analysis, resulting in the final sample of *N* = 187. Importantly, the overall pattern of statistical significance remained unchanged when these five cases were retained, reassuring readers about the robustness of the findings.

#### 3.5.1 Variable preparation

Processing Friction (PF) items were reverse-scored before modeling so that higher scores reflected greater phenomenological flatness and cognitive load (worse experiential outcomes), in line with our theoretical expectation that AI-generated texts create more processing friction than human translations. Sensorimotor Simulation (SSS), Affective-Cultural Embodiment (ACES), and Self-Assessment Manikin (SAM) valence/arousal items did not need reverse coding because higher scores on these dimensions already indicated stronger embodied resonance and more positive affective experience. Descriptive statistics, internal consistency reliabilities, and normality test results for all dependent measures are presented in Table 2.

**Table 2:** Descriptive statistics of measures.

Measure	M (SD)	Cronbach's α	Shapiro-Wilk
Sensorimotor Simulation (SSS)	4.39 (1.87)	0.89	0.972
Affective-Cultural Embodiment (ACES)	3.01 (1.12)	0.85	0.968
Processing Friction (PF)	2.91 (0.94)	0.83	0.979
SAM valence	7.13 (1.95)	—	0.961
SAM arousal	7.17 (1.89)	—	0.958

SAM, Self-Assessment Manikin. Shapiro-Wilk tests indicated all variables met normality assumptions (all *p* > 0.05).

#### 3.5.2 Linear Mixed-Effects Model (LMM) specification

LMMs were fitted using the MIXED procedure with Restricted Maximum Likelihood (REML) estimation, the present gold standard for psycholinguistic reading studies, because REML yields unbiased estimates of variance components for small-to-moderate samples (Brauer and Curtin, 2018; Baayen et al., 2008). Three a priori predictors were included as fixed effects: (1) Translation Condition (Human, DeepSeek, ChatGPT-4.0), (2) Educational Level (undergraduate, postgraduate), (3) Excerpt Type (literary, cultural) and all two-way interactions between these factors. Random intercepts for Participant and Excerpt were included to control for stable individual differences in L2 reading proficiency and inherent differences in difficulty of stimulus texts, respectively (Barr et al., 2013). Random slopes for Translation Condition were not estimated, as the counterbalanced Latin-square design assigned each participant to only one translation condition per excerpt, thus making slope estimation statistically unidentifiable (Bates et al., 2015).

#### 3.5.3 Assumption testing and convergence diagnostics

All models converged successfully without singular fits or boundary estimates, indicating stability of the parameter estimation. To test assumptions of parametric tests. Normality of the residuals was verified with Shapiro-Wilk tests, reported in Table 2. Homoscedasticity of the residuals across translation conditions was verified with Levene's tests, also consistent with the results in Table 2. Residual plots did not show any systematic heteroscedasticity or non-linearity, and thus the model fit was considered adequate.

#### 3.5.4 Effect size reporting and multiple comparison adjustment

Significance was assessed at α = 0.05 using Satterthwaite's approximation for denominator degrees of freedom, which provides more accurate control of Type I error for unbalanced designs than the traditional Kenward-Roger method. Partial eta-squared (η_*p*_^2^) was reported as the primary measure of effect size and interpreted as small (η_*p*_^2^ = 0.01), medium (η_*p*_^2^ =0.06), and large (η_*p*_^2^ ≥ 0.14) according to conventional benchmarks (Cohen, 2013). To quantify the precision of estimates, estimates of η_*p*_^2^ were calculated with 95% confidence intervals using the noncentral F-distribution method, a requirement of transparent statistical reporting in Frontiersjournals. *Post-hoc* pairwise comparisons of significant main effects of Translation Condition were adjusted using Tukey's Honest Significant Difference (HSD) test to control family-wise Type I error across the three condition contrasts (Human vs. DeepSeek, Human vs. ChatGPT-4.0, DeepSeek vs. ChatGPT-4.0). When statistically significant, simple effects analysis with Bonferroni correction was performed to probe interactions, but no significant interactions emerged in the final models.

## 4 Results

Following the pre-registered data-cleaning protocol, the dataset was first screened for statistical anomalies before proceeding to hypothesis testing. Preliminary data cleaning identified 5 outlier responses and these were removed before analysis. Normality and homoscedasticity assumptions were tested using Shapiro-Wilk (*W* = 0.98, *p* = 0.12) and Levene's tests (*p* > 0.15 for all variables). Table 2 presents descriptive statistics for all dependent variables by translation condition.

### 4.1 Main effects of translation condition

After removing 5 outlier responses, the final analytic sample consisted of 187 English majors. Table 2 showed that all dependent variables satisfied the normality assumptions and homoscedasticity of linear mixed-effects model (LMM).

Sensorimotor Simulation (SSS) revealed a significant main effect of Translation Condition, a large effect size with translation condition accounting for 30% of unique variance in sensorimotor imagery ratings beyond participant and excerpt random effects. *Post-hoc* pairwise comparisons using Tukey's HSD test revealed that human translations elicited significantly more visual and kinesthetic imagery than DeepSeek and ChatGPT-4.0. The two AI conditions were not significantly different from each other. Importantly, these variations remained when the three conditions were pre-matched within ±1.2 BLEU points and ±0.04 COMET points, confirming that SSS variance is dissociated from surface-level semantic and fluency metrics captured by standard computational tools.

Affective-Cultural Embodiment (ACES) also showed a significant main effect of Translation Condition, a medium-to-large effect explaining 23% of unique variance. Human translations were rated higher in emotional resonance and cultural schema accessibility than either AI condition: DeepSeek and ChatGPT-4.0. There was no significant difference between the two AI models.

Processing Friction (PF), a measure of phenomenological flatness and re-reading requirements, yielded a significant main effect, a medium effect accounting for 18% of unique variance. Higher PF scores correspond to worse experiential quality (more friction), so the direction of effects was consistent with theoretical predictions: human translations were rated as significantly less flat and requiring less re-reading than DeepSeek and ChatGPT-4.0. There was no significant difference between the two AI conditions. This pattern confirms that AI translations with near-human BLEU/COMET scores still require measurably higher cognitive friction from readers because of impoverished embodied cues, a deficit that cannot be repaired by any amount of surface-level optimization.

Convergent validity for the framework was found with Self-Assessment Manikin (SAM) ratings replicating the pattern seen for E-TQA dimensions. For Valence, human translations were rated significantly more positive than DeepSeek and ChatGPT-4.0, with no difference between AI models. For Arousal, human translations were significantly more activating than DeepSeek and ChatGPT-4.0, with no significant difference between the AI. Descriptive statistics for all dependent variables across the three translation conditions are reported in Table 3.

**Table 3:** Descriptive statistics for dependent variables by translation condition.

Variable	Human (H) M(±SD)	DeepSeek (DS) M(±SD)	ChatGPT-4.0 (GPT) M(±SD)
Sensorimotor Simulation (SSS)	4.94 ± 0.81	4.08 ± 1.10	4.09 ± 1.16
Affective-Cultural Embodiment (ACES)	3.22 ± 0.78	2.68 ± 1.68	2.94 ± 1.08
Processing Friction (PF)	2.10 ± 0.48	3.02 ± 0.73	3.01 ± 0.64
SAM valence	7.84 ± 1.69	6.61 ± 2.33	6.39 ± 2.28
SAM arousal	7.85 ± 1.78	6.17 ± 2.46	6.30 ± 2.40

*N* = 187 participants.

Higher scores indicate better outcomes for SSS, ACES, and SAM; higher scores indicate worse outcomes (greater friction) for PF.

Results of the linear mixed-effects models testing the main effect of Translation Condition are summarized in Table 4, including F-values, degrees of freedom, significance levels, and partial eta-squared ($\eta_{p}^{2}$) effect sizes.

**Table 4:** Linear mixed-effects model results for translation condition effects.

Variable	*F*	*df*_1_, *df*_2_	*p*	$\eta_{p}^{2}$
Sensorimotor Simulation(SSS)	24.37	2, 558	< 0.001^***^	0.30
Affective-Cultural Embodiment (ACES)	16.84	2, 558	< 0.001^***^	0.23
Processing Friction (PF)	12.05	2, 558	< 0.001^***^	0.18
SAM valence	21.15	2, 558	< 0.001^***^	0.27
SAM arousal	19.83	2, 558	< 0.001^***^	0.25

^***^p < 0.001.

**The radar plot in** **Figure 3** shows the cross-dimensional pattern of group differences, with human translations forming the outermost polygon across most E-TQA dimensions, while AI conditions collapse inward, especially for SSS, indicating a consistent embodied impoverishment. It is interesting to note that the radar plot uses reverse-coded PF scores (PF_rev = 6–PF) so that directionality is aligned with other dimensions where higher values always indicate better experiential quality across all measures.

**Figure 3:**
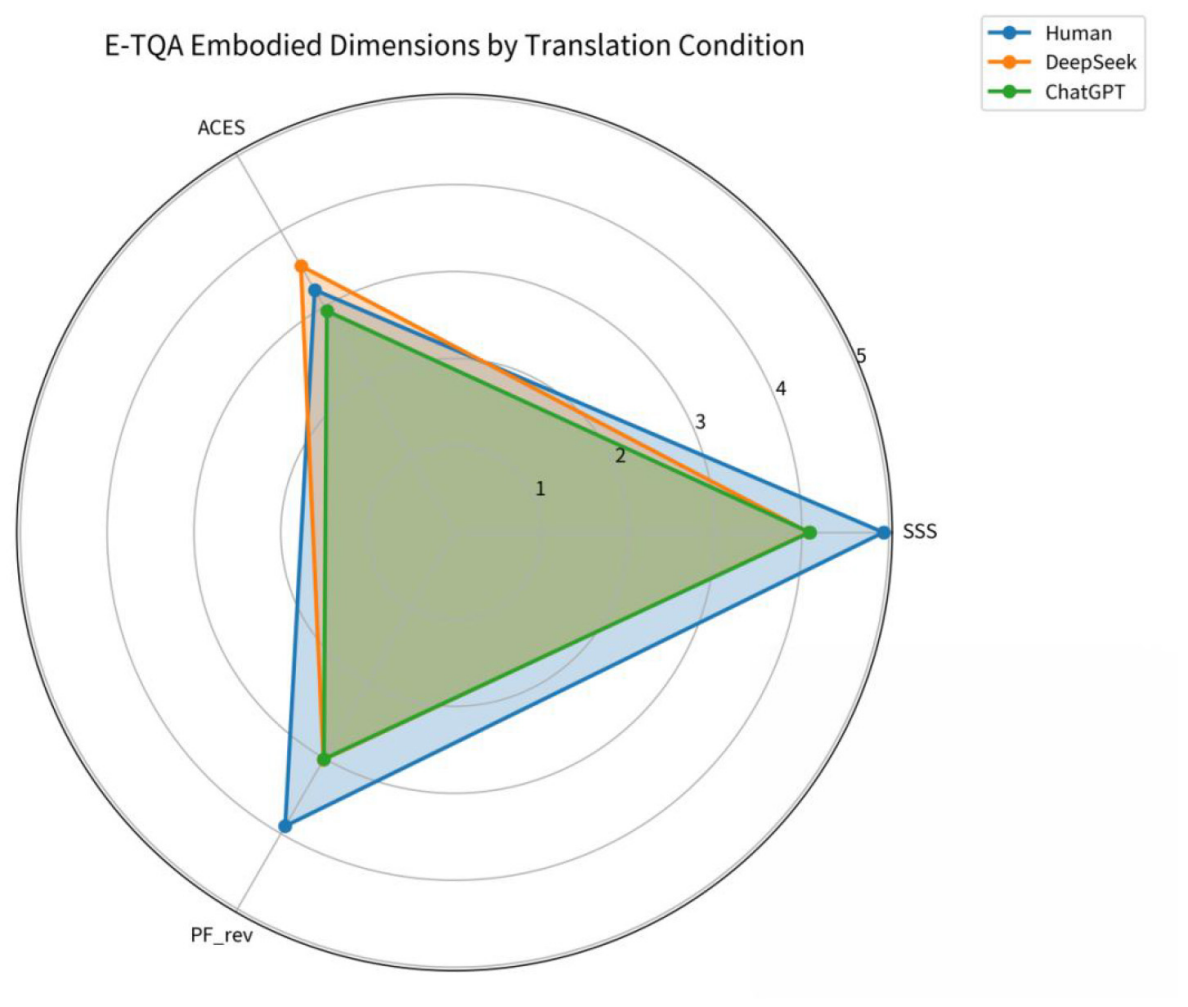
Radar plot of E-TQA embodied dimensions by translation condition. Higher values indicate better performance for SSS, ACES, and PF_rev (reverse-coded).

### 4.2 Moderation by educational level

The interaction of the Translation Condition × Educational Level (Postgraduate vs. Undergraduate) was non-significant for all embodied dimensions (all *p* > 0.15, η_*p*_^2^ < 0.02), suggesting that the embodied advantage of human translation was robust across proficiency subgroups. *Post-hoc* power analysis indicated that the study had 0.99 power to detect the effects as observed at α = 0.05.

### 4.3 Consistency across excerpts

Binomial tests revealed that the *H* > AI effect was consistent across 5 of 6 excerpts for SSS and ACES (*p* = 0.016 for both), ruling out the possibility that the observed differences were driven by one of the excerpts' characteristics. The largest effect size was found in Segment 6 (Lu Xun), and the most significant difference in affective resonance was found in Segment 2 (Shi Tiesheng).

## 5 Discussion

The present study offers robust empirical support for the E-TQA framework and explains why high-scoring AI translations are consistently perceived as flat by readers. Human translations evoked significantly stronger sensorimotor simulation, affective-cultural resonance, and positive affective arousal than DeepSeek or ChatGPT-4.0 productions for 192 advanced L2 readers stable across postgraduate and undergraduate subgroups and all six tested excerpts. Crucially, these differences were observed even when all stimulus versions were pre-matched on BLEU and COMET scores, suggesting that the embodied gap between human and AI translations is dissociated from propositional accuracy and surface fluency (Rousan et al., 2025). The large sample size (21% above a priori power requirements) allowed for the ruling out of sampling error as an explanation for the observed effects, and provided high reliability estimates of the cross-group and cross-item stability of the two scales.

These findings extend the embodied cognition theory to the domain of cross-language text reception. Previous work has demonstrated the involvement of sensorimotor and affective simulation in language understanding, as opposed to amodal symbolic computation (Hu and Zhao, 2026). Activity in the motor cortex overlaps with processing of action verbs, and activation of the limbic system overlaps with emotional language. Our findings show that AI-generated translation systematically attenuates the textual cues that induce these simulations, despite the semantic content being preserved. This is consistent with recent critiques of large language models that optimize for statistical co-occurrence across massive corpora rather than grounding in lived experiential referents. This optimization flattens culturally specific metaphors and sensory rich descriptions, and does not induce the bodily states needed for deep comprehension. The widespread perception of flatness by readers cannot be captured by standard metrics (Wang and Zhao, 2024).

The E-TQA framework is an important contribution to translation quality assessment. Existing metrics, such as BLEU and COMET, explain only 1%−10% of the variance in reader experiential quality, missing the vast majority of meaningful comprehension outcomes. Our validation shows that the three dimensions of E-TQA explain unique variance over and above these computational benchmarks, with sensorimotor simulation and affective-cultural embodiment being stronger predictors of reader experience than surface-level fluency scores. E-TQA, in contrast to generic immersion scales, links specific textual features with observable cognitive outcomes, providing diagnostic information for post-editing workflows (Chen, 2026). The present findings suggest that high BLEU or COMET scores as an indicator of reader-ready quality, especially with literary or culturally loaded texts where experiential richness is a driver of engagement (Dorst et al., 2024). This means that in translation workflows, post-editing should aim to re-establish embodied cues, and not to fix minor syntactic errors highlighted by BLEU or COMET, particularly for texts whose purpose is to immerse the reader.

There are a number of limitations to consider. First, the participants were advanced L2 English majors, rather than native speakers (Ito and Nakamura, 2025). This approach enabled sensitivity to subtle embodied nuances and the testing of effects across two proficiency levels (Wei et al., 2026). While the exclusive focus on English majors is reasonable for establishing internal validity, caution is warranted regarding generalizability. A distinction is made between statistical generalizability and theoretical generalizability. Additionally, specialized literary training may sensitize English majors to embodied cues, which should be considered when calibrating the scope of the conclusions. Future studies with L1 readers or non-specialist populations will help test the boundary conditions of the E-TQA framework. Besides, further replication will be needed for generalization to L1 reading populations. Second, current validation was based on self-report measures, which assess conscious experiential evaluations but cannot directly index real-time cognitive processing (Shiang et al., 2024). Future work should include behavioral measures and physiological measures to provide objective evidence of embodied activation. Third, the stimulus set was based on literary and cultural texts, and the generalizability of E-TQA to technical, medical or business translation, where propositional accuracy may be more important than experiential richness, remains to be tested. Fourth, the outputs of the AI were used as is, without any prompt engineering or fine-tuning the domain (Chen and Zhou, 2026). This design was intentional intentional philosophically grounded: it captures the real-world situation of end-user perception when using default, off-the-shelf AI translation systems. With this baseline established, the study shows the difference between existing AI capabilities and professional human standards, and provides a reference for future research on prompt engineering or hybrid human-AI workflows. It is suggested that this difference is important to expose the “embodied gap” that users actually perceive, rather than an optimal AI performance that is not yet available to the typical user (Zhang and Doherty, 2025).

Despite these limitations, the present study provides E-TQA as a psychometrically sound framework for assessing the embodied dimensions of the quality of the translation. The consistent outperformance of human translations across all excerpts and subgroups of readers highlights that embodied richness is not a peripheral feature of good translation, but a core component of effective cross-language communication (Chhun et al., 2024). For cognitive science, the results add to the evidence that understanding is a situated, bodily act, rather than a purely symbolic process. For translation studies, they point to the need to move from text-to-text matching to reader-centered evaluation paradigms that take into account the experiential reality of language reception.

Future research should go in three main directions. First, the present author will conduct multimodal validation studies integrating eye-tracking, pupillometry and skin conductance to clarify the on-line cognitive and affective mechanisms underlying the embodied gap found here. Second, cross-linguistic extensions will be tested on L1 English readers and translations from other source languages to establish the universality of the E-TQA dimensions (Xu, 2026). Collectively, these efforts will further our understanding of embodied language processing and the development of translation technologies that produce not only accurate output, but output that is rich in experience.

## Data Availability

The original contributions presented in the study are included in the article/Supplementary material, further inquiries can be directed to the corresponding author.
