# Supplementary material for: When AI translations fall flat: embodied simulation and effort in cross-language reading
Source: Front Psychol. 2026 Oct 7;17:1915913. doi: 10.3389/fpsyg.2026.1915913 (PMC13684984; doi:10.3389/fpsyg.2026.1915913)
Supplement: Supplementary file 1 [file Supplementary_file_1.docx]

**Supplementary Table S1. Latin-Square Allocation of Excerpts to Translation Conditions**

| Segment | Group A | Group B | Group C |
| --- | --- | --- | --- |
| 1. Bing Xin, The Little Orange Lamp | H | DS | GPT |
| 2. Shi Tiesheng, The Temple of Earth and I | DS | GPT | H |
| 3. Lu Xun, Medicine | GPT | H | DS |
| 4. Ba Jin, In Memory of Xiao Shan | H | GPT | DS |
| 5. Yu Dafu, Autumn in Peiping | DS | H | GPT |
| 6. Lu Xun, In Memory of Miss Liu Hezhen | GPT | DS | H |

Note. H = human translation, DS = DeepSeek-default, GPT = ChatGPT-4.0-default.

**Supplementary Table S2. Latin-Square Allocation of Excerpts to Translation Conditions (coded)**

| Segment | Group A | Group B | Group C |
| --- | --- | --- | --- |
| 1. Bing Xin, The Little Orange Lamp | SEG1&1 | SEG1&2 | SEG1&3 |
| 2. Shi Tiesheng, The Temple of Earth and I | SEG2&2 | SEG2&3 | SEG2&1 |
| 3. Lu Xun, Medicine | SEG3&3 | SEG3&1 | SEG3&2 |
| 4. Ba Jin, In Memory of Xiao Shan | SEG4&1 | SEG4&3 | SEG4&2 |
| 5. Yu Dafu, Autumn in Peiping | SEG5&2 | SEG5&1 | SEG5&3 |
| 6. Lu Xun, In Memory of Miss Liu Hezhen | SEG6&3 | SEG6&2 | SEG6&1 |

**Segments:**

**SEG1&1**

I carried this dainty little orange lamp, walking slowly along the dark and moist mountain road. The dim orange light from it could barely light up the path for any distance (Bing, 1957/2017). (The Little Orange Lamp, Bing Xin, Trans. Lei Jing, 2017)

**SEG1&2**

DeepSeek: I carried the nimble little tangerine lamp and walked slowly along the dark, damp mountain path. The hazy reddish-orange glow couldn’t really reach very far.

**SEG1&3**

ChatGPT 4.0: I carried the delicate little orange lamp and walked slowly along the dark, damp mountain path. The hazy orange-red light really couldn't illuminate very far.

**SEG2&1**

Summer is rows of dazzling, scorching stone benches, or cool stone steps covered in moss, with fruit peels below and a half-crumpled, sat-upon newspaper above (Shi, 1991/2007). (“The Temple of Earth and I”, Shi Tiesheng, Trans. Karen Gernant & Chen Zeping, 2007)

**SEG2&2**

DeepSeek: In summer, there are dazzling, scorching stone benches, or cool stone steps covered in moss, with fruit peels under the steps and a crumpled, half-sat-upon newspaper on top of them.

**SEG2&3**

ChatGPT 4.0: Summer is composed of rows of dazzling and scorching stone benches, or shady stone steps covered in moss. Below the steps, there are fruit peels, and on the steps, there is half a newspaper, crumpled from being sat on.

**SEG3&1**

He walks out into the street, sits down under the grey, cold glow of the lamp, pulls out the silver dollar, kneads it over and over, until his hand is slick with sweat (Lu, 1919/1990). (“Medicine”, Lu Xun, Trans. William A. Lyell, 1990)

**SEG3&2**

DeepSeek: Old Shuan went out into the street. He sat down under the pale, cold lamplight, took out that silver dollar, and squeezed it again and again until it was covered with sweat.

**SEG3&3**

ChatGPT 4.0: Hua Laosan walked onto the street, sat under the dim, cold light, took out the silver dollar, squeezed it repeatedly, and found his hands sweaty.

**SEG4&1**

No tears came to my eyes, but I felt an agony as if thousands of sharp nails were pricking and scratching at my heart (Ba, 1979/1988). (“In Memory of Xiao Shan”, Ba Jin, Trans. Jock Hoe, 1988)

**SEG4&2**

DeepSeek: I didn't shed any tears, but I felt as if countless sharp fingernails were clawing at my heart.

**SEG4&3**

ChatGPT 4.0: I didn’t shed tears, but I felt as if countless sharp nails were scratching at my heart.

**SEG5&1**

Autumn in the South also has its unique features, such as the moonlit Ershisi Bridge in Yangzhou, the flowing sea tide at the Qiantangjiang River, the mist-shrouded Putuo Mountain and lotuses at the Lizhiwan Bay. But they all lack strong colour and lingering flavour. Southern autumn is to Northern autumn what yellow rice wine is to kaoliang wine, congee to steamed buns, perches to crabs, yellow dogs to camels (Yu, 1934/2007). (“Autumn in Peiping”, Yu Dafu, Trans. Zhang Peiji, 2007)

**SEG5&2**

DeepSeek: The luminous moon over the Twenty-Four Bridges, the autumn tide of the Qiantang River, the cool mist on Mount Putuo, and the withered lotus on Litchi Bay are all lacking in intensity of color and lasting aftertaste. Compared with autumn in the north, they are like rice wine versus strong liquor, congee versus steamed buns, perch versus river crab, a yellow hound versus a camel.

**SEG5&3**

ChatGPT 4.0: The bright moon over the Twenty-four Bridges, the autumn tide of the Qiantang River, the cool mist of Mount Putuo, and the withered lotus of Lychee Bay are all somewhat bland in color and lack lasting aftertaste. Compared to the autumn of the northern land, they are like yellow wine to strong spirits, porridge to steamed buns, perch to crabs, and yellow dogs to camels.

**SEG6&1**

Such horrors have made my eyes too sick to see; such slanders have made my ears too pained to hear. What more can I say? I now understand why a dying nation falls silent. Silence! Still silence! If we do not burst out in this silence, we shall perish in it (Lu, 1926/1980). (“In Memory of Miss Liu Hezhen”, Lu Xun, Trans. Yang Xianyi & Gladys Yang, 1980)

**SEG6&2**

DeepSeek: The horrible sights are already too much for my eyes to bear; the rumors are even too much for my ears to take. What more can I say? I see now why a declining nation keeps silent. Silence, silence! If not in silence, then break out; if not in silence, then perish.

**SEG6&3**

ChatGPT 4.0: The tragic sights have become unbearable for my eyes; the rumors are even more intolerable for my ears. What more can I say? I understand now why a declining nation remains silent. Silence, silence! Either a breakout in silence or extinction in silence.
